# High perceived stress and social interaction behaviour among young adults. A study based on objective measures of face-to-face and smartphone interactions

**DOI:** 10.1371/journal.pone.0218429

**Published:** 2019-07-26

**Authors:** Agnete Skovlund Dissing, Tobias Bornakke Jørgensen, Thomas Alexander Gerds, Naja Hulvej Rod, Rikke Lund

**Affiliations:** 1 Section of Social Medicine, Department of Public Health, University of Copenhagen, Copenhagen, Denmark; 2 Section of Epidemiology, Department of Public Health, University of Copenhagen, Copenhagen, Denmark; 3 Department of Sociology, University of Copenhagen, Copenhagen, Denmark; 4 Section of Biostatistics, Department of Public Health, University of Copenhagen, Copenhagen, Denmark; 5 Copenhagen Stress Research Centre, Copenhagen, Denmark; 6 Center for Healthy Aging, Faculty of Health Sciences, University of Copenhagen, Copenhagen, Denmark; University of Zurich, SWITZERLAND

## Abstract

Stress and mental health problems impede social functioning and may also complicate relationship formation with peers. The aim was to investigate whether high perceived stress among young adults is associated with social interaction behaviour both via face-to-face interaction and via smartphone interaction. The data was derived from the Copenhagen Network Study, where 535 first-year students (mean age 21.3, 77% male) self-reported on perceived stress at baseline and were subsequently followed for three months with continuous Bluetooth recordings of face-to-face interactions and smartphone interactions (calls and texts) measuring the network size, frequency, and duration of interactions. Logistic regression was used to assess associations between perceived stress (high/low) and social interactions adjusting for sex, age, and personality traits. Participants with high perceived stress were more likely to engage in a larger call and text network and have a higher call and text frequency compared to individuals with low perceived stress. We found a non-statistically significant tendency that participants with a high perceived stress level spend less time meeting face to face with peers. Stressed students engage in frequent smartphone interaction which may be explained by a social support seeking behaviour, or it may be that accommodating a large network via the smartphone is stress-inducing.

## Introduction

Engaging in social interaction is a key aspect of establishing social relations and taking part in social interaction increases well-being and has health benefits [1–5]. Engaging in social interaction with peers is especially important to young adults, who are in a transitional process of establishing lives of their own and gaining increasing independence from their parents [6]. However, in this important period of life, many young adults suffer from stress and poor mental health [7–10]. Recent figures reveal an increase in perceived stress among Danish young adults. In 2013, 24% of 16-24-year-olds reported high perceived stress. In 2017, the figure had risen to 32% among the same age group [11]. Perceived stress and poor mental health are also prevalent in university populations outside Denmark [9, 10]. Stress and poor mental health in early life have been shown to be related to anti-social behaviour and poor social relations in later adulthood [12, 13], and it is possible that young adults with high perceived stress also have difficulties engaging in social interactions and establishing social relations.

Perceived stress is defined as feelings of unpredictability in one’s life situation, feeling unable to manage everyday challenges, and feeling that one’s problems keep piling up [14]. Perceived high and prolonged stress can manifest itself in an array of symptoms, including anxiety, depressive symptoms, and fatigue [15]. These stress-related symptoms may also hamper social interaction. A review study has suggested that individuals with depressive symptoms suffer from reduced social functioning, such as poorer control of their feelings, reduced ability to concentrate on the topic of conversation, increased self-focus, and less smiling during social interaction [16]. As perceived stress and depressive symptoms are closely related [17], some of the same mechanisms may also occur in relation to stress, possibly leading to withdrawal from peers. A few studies [12, 18] have investigated stress in relation to the number of self-reported social relationships which suggested that indicators of stress are related to low maintenance of social relationships. However, in these studies it is likely that the level of stress affects how respondents perceive and hence self-report on social relationships, possibly leaving bias in the result. One study using an objective measurement method also found that a high level of perceived stress was related to a low number of face-to-face interactions, but the study was conducted on a small population of fewer than 70 participants [19].

Interacting via smartphones continues to be an important aspect of young adults’ social interaction behaviour. The smartphone is an important tool used to keep in contact with friends, family and extended networks, and a large proportion of social interaction among young adults takes place via smartphones [20, 21]. Young adults place on average 10 calls per day, and approximately 18% of young adults send more than 200 text messages a day [22]. The smartphone, which is readily accessible when needed, is an easy and less confrontional mode of interaction, enabling immediate disclosure of negative emotions, and may therefore be used as tool to alleviate distress [23, 24]. Two qualitative studies conducted among university students suggested that smartphones are used to seek social support [25, 26]. In relation to perceived stress, seeking social support using smartphones can possibly lead to a high frequency of interaction if network members are available. Even though smartphone interactions among young adults are very common, studies investigating how high perceived stress might affect smartphone interaction behaviour are sparse, and the few findings that exist are mixed, suggesting both higher and lower numbers of smartphone interactions in relation to high stress [19, 23, 24, 27]. Further, none of these studies accounted for personality traits, which are strong factors in determining both smartphone behaviour [28] and perceived stress [29].

Hence, further research is needed to investigate the relationship between perceived stress, smartphone interactions and face-to-face interactions among young adults, using objective measurement methods while also accounting for personality factors.

### Aim and hypotheses

In a population of first-year students adapting to a new life situation at university and in the process of establishing new friendships with peers, we investigate whether a high level of perceived stress is related to social interaction behaviour and whether this relationship differ according to face-to-face interactions and smartphone interactions. Based on the current literature, we hypothesize that 1) high perceived stress among young adults is related to fewer face-to-face interactions with peers, and that 2) high perceived stress is related to a high level of smartphone interactions.

## Material and methods

### Study design

We used data from the Copenhagen Network Study (CNS) [30]. 1,333 first-year students at a Danish university were invited to participate in the study; 584 (44%) first-year students accepted the invitation. Subsequently, second- and third-year students were also invited; here, another 307 students agreed to participate and provided the sufficient information on study variables. Participants completed an online baseline questionnaire including questions concerning perceived stress, age, gender, and personality traits. After completing the baseline questionnaire, participants received a smartphone (LG Nexus 4), which ran customized software that recorded all outgoing and incoming calls and text messages assigned a unique identifier for each contacted person (alters). The smartphone also recorded face-to-face encounters among participating students via embedded Bluetooth sensors. To ensure that participants used the distributed smartphone as their primary tool for communication, technical personnel on campus helped the students insert their private SIM cards into the smartphone. Students were recruited continuously throughout the academic year and were followed for three months after responding to the baseline questionnaire, during which smartphone data were continuously collected. Among the 584 first-year students, we excluded 49 individuals with missing information on the perceived stress variable and co-variates leaving 535 participants. Another 123 and 146 participants were further excluded due to missing information on smartphone interactions and Bluetooth-recorded face-to-face interactions, respectively. In total, 412 students were included in the analyses of smartphone interactions, and 389 participants in the analyses of Bluetooth recorded face-to-face interactions.

### Measurements

#### Smartphone interactions

The *network size of interactions* for each participant was calculated by counting the number of unique alters that the participant had interacted with using either calls or texts at least once during the three-month follow-up period. The *frequency of interactions* was calculated by counting the number of calls and texts during the follow-up period. The *duration of interactions* was considered by calculating the average duration of phone calls in minutes (excluding missed calls). The derived smartphone interaction variables showed heavy right-tailed distributions, and hence for use in further analyses the variables were categorized in tertiles using the 33rd and the 66th percentile as cut-points corresponding to low, intermediate, and high levels of smartphone interaction. Using the unique identifiers of alters, we were able to separate out interactions with participating fellow students from interactions with individuals who were not participating in the study.

#### Face-to-face interactions

We measured the average *duration of face-to-face interactions* using proximity recordings by Bluetooth sensors. The Bluetooth software installed on the provided smartphone was designed to scan every five minutes and to detect other smartphones within a distance of approximately 0–10 meters. The Bluetooth function was designed to re-start automatically if participants turned the function off. The received signal strength indicator (RSSI) recorded in each Bluetooth scan was used to calculate the distance between two smartphones. From this information, we were able to consider scans of a maximum distance of two metres between devices, which has been noted as a typical distance in face-to-face interactions [31]. In order to reduce the risk of casual scans not reflecting face-to-face interactions, we restricted the data to encounters lasting at least five minutes. The technique of inferring face-to-face interactions from Bluetooth scans is still in its infancy, but a few studies have showed that Bluetooth-recorded face-to-face interactions are correlated with online friendships [32] and self-reported social interaction [33]. The Bluetooth sensor has been shown to detect other devices in a relatively stable manner even when carried in bags and pockets [32].

#### Perceived stress

Perceived stress was measured using a Danish consensus translation of the Perceived Stress Scale (PSS), originally developed among a population of university students [14]. The 10-item PSS instrument was designed to measure the degree to which everyday situations are appraised as being stressful, measured using a score ranging from 0 to 40. The Danish consensus translation of the PSS has shown good reliability, internal consistency (ICC = 0.87, Cronbach’s alfa = 0.84) and validity [34]. As we aimed at studying participants with the highest stress levels, we defined high stress as the 10% scoring highest on the PSS, corresponding to a cut-off at 20 on the scale. A relative cut-off on the PSS has been used before to identify individuals with high levels of stress [11].

#### Co-variates

Gender, age, and personality were identified in the literature review as confounding variables since they are related to both stress and social interaction behaviour [35–38]. Personality was measured using the 44-item version of the Big Five Inventory, which evaluates five personality traits: *agreeableness*, *conscientiousness*, *extroversion*, *openness*, and *neuroticism*. These five personality sub-scales have shown good internal consistency (Cronbach’s alfa <0.8) and validity [39]. The personality scales were computed on a scale ranging from 1–5 according to guidelines for the 44-item version of the Big Five Inventory and were used in the analyses as continuous scales.

### Analytical strategy

We investigated distributions of perceived stress, co-variates, and the social interaction variables; smartphone interactions and face-to-face interactions. Odds ratios (ORs) with 95% confidence intervals (95% CIs) for the association between perceived stress and the social interaction variables were estimated with logistic regression using the highest tertiles of the social interaction variable as the outcome category. Models were adjusted for identified confounders; age and gender in one model and then further adjusted for personality traits in a final model. In order to evaluate whether smartphone interaction behaviour was different among newly acquainted peers, we divided the analyses into smartphone interactions with participating fellow students and interactions with non-participants. Bluetooth scans of face-to-face interactions were only recorded with participating fellow students.

The following sub- and sensitivity analyses were conducted: 1) First-year students had considerably more compulsory groupwork activities than second- and third-year students at the specific university, and hence it is possible that highly stressed first-year students would withdraw from social life at university to a lesser extent, as hypothesized, had the activities not been compulsory. To evaluate results concerning Bluetooth face-to-face interactions that reflect compulsory interactions to a lesser extent, we included second- and third-year students (N = 307) in an additional analysis. (2) To reduce the risk of counting service calls and the like, we restricted the network size of calls interactions to counting the number of alters called at least three times. (3) We conducted an analysis using the perceived stress scale as continuous and a log-transformed version of the continuous smartphone interaction variables in linear regression models. Results from this analysis are not straightforward to interpret but give an indication of whether the main results are robust to the categorisation of the interaction variables using tertiles. Statistical computations were carried out with R version 3.2.4.

### Ethics and privacy

All participants gave informed consent and were able to withdraw from the study at any time. All data were used anonymously, and the Copenhagen Network Study was approved by the Danish Data Protection Agency (approval number: 2012-41-0664).

## Results

### Characteristics of the study population

The majority of the population was comprised of men (76.8%) and the mean age was 21.3 years (range 18–42 years), which is in line with the average first-year student at that specific university (68% male, mean age 21). During the three-month period, we observed a total of 94,870 phone calls, 615,152 text messages, and 417,000 face-to-face meetings recorded with the Bluetooth sensor. Table 1 shows that the majority (45%) of participants reporting high levels of stress were in the lowest tertile of duration of face-to-face interactions. The opposite pattern appeared for call and text interactions, where between 42% and 61% of those reporting high levels of stress were in the highest tertile of social interactions with respect to social network size, interaction frequency, and call duration. Being woman and scoring low on the personality traits agreeableness and conscientiousness as well as scoring high on neuroticism were strongly associated with reporting high stress (Table 1).

**Table 1:** Associations between gender, age, personality, smartphone interactions, face-to-face interactions, and perceived stress in a population of 535 first-year students.

	Total population	Low stress	High stress	p-value
**Male** N (%)	411 (76.8)	382 (78.1)	29 (63.0)	0.033
**Age** mean (SD)	21.3 (2.7)	21.3 (2.7)	21.5 (2.3)	0.58
**Neuroticism** mean (SD)	2.4 (0.6)	2.4 (0.6)	3.1 (0.6)	<0.001
**Agreeableness** mean (SD)	3.8 (0.4)	3.8 (0.4)	3.7 (0.4)	0.007
**Conscientiousness** mean (SD)	3.5 (0.6)	3.5 (0.6)	3.3 (0.6)	0.035
**Extroversion** mean (SD)	3.4 (0.7)	3.4 (0.7)	3.2 (0.3)	0.13
**Openness** mean (SD)	3.6 (0.5)	3.6 (0.5)	3.7 (0.4)	0.32
**Call network size (number of alters)**^**a**^ N (%)				
1st tertile (0–23)	141 (34.2)	136 (35.9)	5 (15.2)	
2nd tertile (24–36)	141 (34.2)	131 (34.6)	10 (30.3)	
3rd tertile (37–148)	130 (31.6)	112 (29.6)	18 (54.5)	0.007
Missing	123			
**Text network size (number of alters)**^**a**^ N (%)				
1st tertile (5–24)	141 (34.2)	132 (34.8)	9 (27.3)	
2nd tertile (25–38)	145 (35.2)	137 (36.1)	8 (24.2)	
3rd tertile (39–85)	126 (30.6)	110 (29.0)	16 (48.5)	0.064
Missing	123			
**Frequency of call interactions**^**a**^ N (%)				
1st tertile (0–127)	139 (33.7)	136 (35.9)	3 (9.1)	
2nd tertile (126–250)	137 (33.3)	127 (33.5)	10 (30.3)	
3rd tertile (251–1555)	136 (33.0)	116 (30.6)	20 (60.6)	0.001
Missing	123			
**Frequency of text interactions**^**a**^ N (%)				
1st tertile (25–635)	138 (33.5)	130 (34.3)	8 (24.2)	
2nd tertile (636–1570)	137 (33.3)	128 (33.8)	9 (27.3)	
3rd tertile (1571–12,372)	137 (33.3)	121 (31.9)	16 (48.5)	0.15
Missing	123			
**Mean duration in minutes per call**^**a**^ N (%)				
1st tertile (<0–1.60)	137 (33.3)	130 (34.4)	7 (21.2)	
2nd tertile (1.61–2.60)	137 (33.3)	125 (33.1)	12 (36.4)	
3rd tertile (2.61–15.00)	137 (33.3)	123 (32.5)	14 (42.4)	0.28
Missing	124			
**Mean duration in minutes per face-to-face meeting**^**b**^ N (%)				
1st tertile (5–24)	151 (38.8)	137 (38.3)	14 (45.2)	
2nd tertile (25–29)	117 (30.1)	107 (29.9)	10 (32.3)	
3rd tertile (30–84)	121 (31.1)	114 (31.8)	7 (22.6)	0.55
Missing	146			

SD = standard deviation.

^a^ All interactions recorded during the three months follow-up.

^b^ Interactions with participating fellow students recorded during the three months follow-up.

### Perceived stress and smartphone interactions

**Fig 1:**
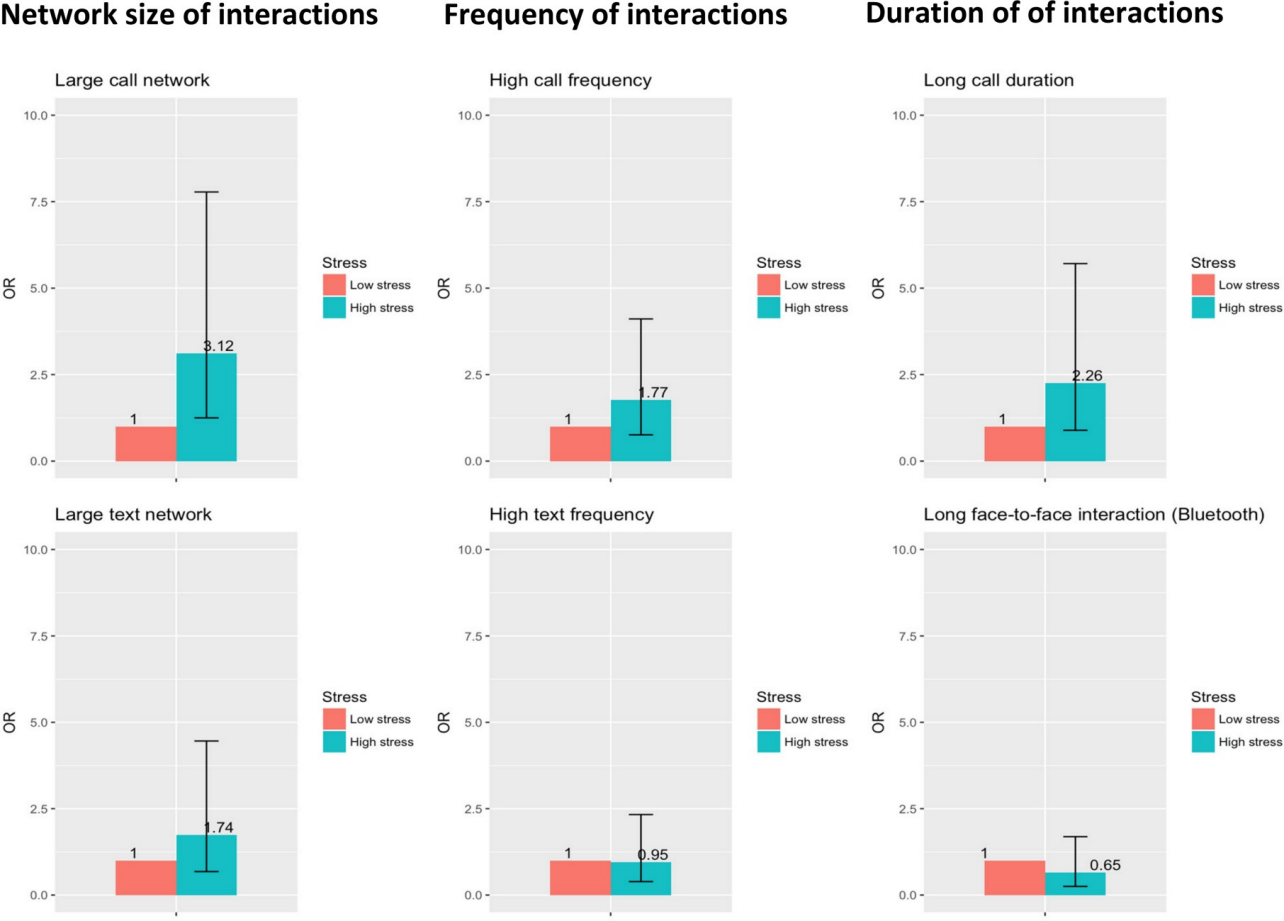
Associations between perceived stress and social interaction with participating fellow students. Adjusted odds ratios for having large call & text networks, high frequency of text & call interactions, and long duration of call and face-to-face interactions among participants with high perceived stress compared to low.

As regards smartphone interactions with participating fellow students, the odds of having a large call network were approximately three times higher for students with high levels of perceived stress (OR = 3.12, 95% CI: 1.25–7.78) than for students with low levels of stress. The same tendency appeared with regard to call frequency (OR = 1.77, 95% CI: 0.76–4.11) and call duration (OR = 2.26, 95% CI: 0.89–5.71), but these differences were not statistically significant (Table 2 and Fig 1).

**Table 2:** Odds ratios and 95% confidence intervals for associations between perceived stress and smartphone interactions in a population of 412 first-year students divided by interactions with participating fellow students and all interactions.

	Total population	Highest tertile of call network		Highest tertile of text network		Highest tertile of call interaction frequency		Highest tertile of text interaction frequency		Highest tertile of call duration		Highest tertile of face-to-face interaction duration^a^	
***Interactions with participating fellow students***													
***Age and gender adjusted***	*** ***	*** ***	*** ***	*** ***	*** ***	*** ***	*** ***	*** ***	*** ***	*** ***	*** ***	*** ***	*** ***
** **	**N (%)**	**OR**	**95% CI**	**OR**	**95% CI**	**OR**	**95% CI**	**OR**	**95% CI**	**OR**	**95% CI**	**OR**	**95% CI**
Low perceived stress	379 (92.0)	1	[Ref]	1	[Ref]	1	[Ref]	1	[Ref]	1	[Ref]	1	[Ref]
High perceived stress	33 (8.0)	2.25*	[1.02–4.96]	1.41	[0.62–3.21]	1.49	[0.70–3.14]	0.83	[0.37–1.87]	2.13	[0.91–4.96]	0.59	[0.24–1.42]
***Age*, *gender and personality adjusted***		*** ***	*** ***	*** ***	*** ***	*** ***	*** ***	*** ***	*** ***	*** ***	*** ***	*** ***	*** ***
** **	**N (%)**	**OR**	**95% CI**	**OR**	**95% CI**	**OR**	**95% CI**	**OR**	**95% CI**	**OR**	**95% CI**	**OR**	**95% CI**
Low perceived stress	379 (92.0)	1	[Ref]	1	[Ref]	1	[Ref]	1	[Ref]	1	[Ref]	1	[Ref]
High perceived stress	33 (8.0)	3.12*	[1.25–7.78]	1.74	[0.68–4.46]	1.77	[0.76–4.11]	0.95	[0.39–2.33]	2.26	[0.89–5.71]	0.65	[0.25–1.69]
***Interactions with non-participants***													
***Age and gender adjusted***	*** ***	*** ***	*** ***	*** ***	*** ***	*** ***	*** ***	*** ***	*** ***	*** ***	*** ***	*** ***	*** ***
** **	**N (%)**	**OR**	**95% CI**	**OR**	**95% CI**	**OR**	**95% CI**	**OR**	**95% CI**	**OR**	**95% CI**	**OR**	**95% CI**
Low perceived stress	379 (92.0)	1	[Ref]	1	[Ref]	1	[Ref]	1	[Ref]	1	[Ref]	NA	
High perceived stress	33 (8.0)	2.39*	[1.16–4.92]	1.76	[0.85–3.65]	3.48***	[1.66–7.29]	2.29*	[1.10–4.80]	1.37	[0.65–2.89]		
***Age*, *gender and personality adjusted***		*** ***	*** ***	*** ***	*** ***	*** ***	*** ***	*** ***	*** ***	*** ***	*** ***	*** ***	*** ***
** **	**N (%)**	**OR**	**95% CI**	**OR**	**95% CI**	**OR**	**95% CI**	**OR**	**95% CI**	**OR**	**95% CI**	**OR**	**95% CI**
Low perceived stress	379 (92.0)	1	[Ref]	1	[Ref]	1	[Ref]	1	[Ref]	1	[Ref]	NA	
High perceived stress	33 (8.0)	3.22**	[1.36–7.63]	2.28	[0.97–5.35]	4.23***	[1.82–9.80]	3.03**	[1.32–6.99]	1.25	[0.56–2.82]		

OR = odds ratio; 95% CI = 95% confidence interval.

^a^ 23 missing observations in this model.

*p-value < 0.05

**p-value < 0.01

***p-value < 0.001.

Concerning interaction with non-participants, the associations with high perceived stress were more pronounced. Compared to low stress, high stress was related to having a large call network (OR = 3.22, 95% CI: 1.36–7.63), and many call (OR = 4.23, 95% CI: 1.82–9.80) and text message (OR = 3.03, 95% CI:1.32–6.99) interactions (Table 2). Restricting the network size of call interactions with non-participants called at least three times did not change these conclusions, although the estimate increased (OR = 6.01, 95% CI: 2.52–14.37).

Generally, the above-reported estimates were stable when adjusting for age, gender, and personality factors, although the estimates appeared to increase slightly when adjusting for the personality trait neuroticism, possibly caused by neuroticism being negatively associated with smartphone interaction and strongly positively associated with perceived stress. Treating the perceived stress and smartphone interaction variables as continuous in linear regression models did not change the conclusion of the results, although the association between a high level of stress and a large call network of fellow students was less pronounced (S1 Table).

### Perceived stress and face-to-face interactions

Highly stressed individuals tended to spend less time interacting face to face with participating fellow students (OR = 0.65, 95% CI: 0.25–1.69) although the difference was not statistically significant (Table 2 and Fig 1). When including second- and third-year students (for whom social interactions are less likely to be compulsory), this association appeared more pronounced, and highly stressed students were approximately 60% less likely to interact face to face with fellow students (OR = 0.38, 95% CI: 0.19–0.78).

## Discussion

In this comprehensive study which considered both objectively recorded smartphone interactions and face-to-face interactions, we found that, compared to young adults with low levels of stress, highly stressed young adults interacted with a wider range of individuals using the smartphone, and more frequently, while also engaging in smartphone interaction with a wider range of participating fellow students. At the same time, we found some indication that stressed young adults spend less time interacting face to face with peers, a difference that was pronounced when including second- and third-year students.

The use of smartphones to alleviate distress has been examined elsewhere in relation to broader smartphone use, including non-communicative interactions such as searching for information on the internet, and escapist entertainment [40]. The results in the present study suggest that a high perceived level of stress among young adults is also related to a high number of socially driven smartphone interactions: call and text message communication. A study comprising 69 American college students found that students who felt stressed had fewer objectively measured call and text interactions [19] than non-stressed students, which is contrary to our finding. This is surprising, since the same comparable objective measurement methods of smartphone interactions were employed. In a group of 395 American adults, it was found that individuals with depressive symptoms self-reported using their phone as a means to alleviate negative feelings by spending more time on communication activities [24]. Although we did not investigate depressive symptoms, this finding is in line with our results.

A recognized coping strategy when feeling stressed is to seek social support from intimate social relations [15]. It is possible that stressed participants may use their smartphones to contact existing intimate social relations such as parents or friends in order to seek support to cope with their perceived stress [25, 26]. Even though we were unable to tell from the data whether the smartphone interactions were in fact carried out with family members or other close social relations, this interpretation is to some extent supported by the finding that stressed participants had markedly higher smartphone communication activity as regards smartphone interactions with non-participants, both in terms of network size and contact frequency. Contact frequency and network size have often been described as opportunity structures facilitating access to social support [3], and it is possible that social support, such as helping to overcome emotional distress or giving advice, takes place during the smartphone interaction. As we did not have access to the contents of the calls and text interactions, we were unable to directly evaluate whether they involved aspects of social support. Future research investigating stress and smartphone interactions could benefit from collecting information on the content of smartphone communication to further explore social-support-seeking behaviour using mobile phone technology.

We found a tendency that perceived stress was related to spending less time on face-to-face interactions with fellow students detected with Bluetooth sensors but this finding was not statistical significant. Although the we did not find strong evidence to support that high perceived stress is related low levels of face-to-face interactions, the literature suggest some evidence to support that highly stressed individuals may have difficulties engaging in face-to-face interaction and maintaining relationships over time. In a small population of college students, indicators of stress was reported to be related to a low level of Bluetooth-recorded face-to-face interactions [19]. Two other follow-up studies found that distress is related to a decrease in the number of social relationships over time in adults [12], and that a physiological indicator of stress–altered cortisol levels–is related to lower friendship maintenance among college students [18].

### Strengths and limitations

To the best of our knowledge this is the first study to investigate perceived stress in relation to objectively recorded face-to-face and smartphone interactions in a sample of young adults taking into account important confounding factors. It should be noted, however, that we were not able to collect data on all communication platforms in the smartphone, and hence information on some smartphone interactions is missing, e.g. interactions via Snapchat and Messenger.

We collected the information on perceived stress at baseline, and subsequently followed participants for three months during which smartphone interactions were recorded. Even though the measurements of perceived stress and social interaction were collected at separate times, we cannot exclude reverse causality explanations. Having a large social network may induce stress because of the time and energy spent on maintaining social relations or because of a mismatch in expectations due to having many different social roles [41]. In addition, young adults might feel pressured to accommodate an existing large network through constant texting or calling, which may also induce stress [42]. Reverse causality explanations may be most applicable to the analyses of smartphone interactions with non-participants, whereas this explanation may be less relevant to interactions directed at participating fellow students. The study was conducted among newly enrolled first-year students who were unacquainted at enrolment to university. Hence, it is likely that the recorded smartphone interactions directed at participating fellow students reflect the onset of social interactions and thus these social interactions are not likely to precede the level of perceived stress which is measured at baseline close to enrolment. Momentary reports of stress throughout the follow-up period versus stress measured at baseline could have allowed for a more specific evaluation of whether people reached out to others using their smartphone in moments of stress; this design should be considered in future studies.

The results concerning face-to-face interactions recorded with the Bluetooth sensor may be subject to measurement error. It is likely that the Bluetooth sensor did not record all face-to-face interactions, for example at times when participants did not carry the smartphone with them. It is further possible that some Bluetooth recordings reflect proximity of individuals not engaging in face-to-face interaction such as when queuing. We tried to minimize this error by only counting recordings of a considerable duration and within a given relative distance likely for social interaction. As the Bluetooth recordings of face-to-face interactions are made independently from participants’ reports of perceived stress, this non-differential measurement error is most likely to have biased the results towards the null, which could explain the weak associations detected between perceived stress and face-to-face interactions. Deriving face-to-face interactions from Bluetooth scans is a technique still in it’s infancy, and further work should be carried out validating this measurement method against measures such as self-reports, other wearables devices, or online social networks.

Because of the relatively low response rate, selection mechanisms in the study is a concern. Unfortunately, we did not have the necessary data to explore characteristics of non-respondents. Further, it should be noted that the study was conducted within a group of students enrolled in higher education with a high proportion of men, and hence the generalizability of the results may not extend beyond this group of young adults. The communication platforms used for social interaction among young adults are constantly changing, and also differ according to the social context; this should also be considered when generalizing the results of this paper.

### Conclusion

Young adults with perceived high stress tend to be in contact with a wider range of people and have a higher contact frequency via smartphones than those with low perceived stress. The high smartphone interaction may be an expression of social-support-seeking behaviour or a result of maintaining a large social network via the smartphone. Social networks and they ways in which they can be reached through modern technology appear to be closely related to stress levels among young adults.

## Supporting information

S1 Table Linear regression of the associations between continuous perceived stress and logged continuous call&text interaction measures. (DOCX)
