# Supplementary material for: High perceived stress and social interaction behaviour among young adults. A study based on objective measures of face-to-face and smartphone interactions
Source: PLoS One. 2019 Jul 26;14(7):e0218429. doi: 10.1371/journal.pone.0218429 (PMC6660065; doi:10.1371/journal.pone.0218429)
Supplement: S1 Table — (DOCX) [file pone.0218429.s001.docx]

| **S1 Table: Linear regression of the associations between continuous perceived stress (independent variable) and logged continuous call&text interaction measures (dependent variables)** | | | | | | |
| --- | --- | --- | --- | --- | --- | --- |
|  | **Interactions with fellow students** | | | **Interactions with persons outside study** | | |
| **Call&text interactions** | **Coef.** | **95%CI** | **p-value** | **Coef.** | **95%CI** | **p-value** |
| Log (call network) | 0.00 | [-0.01;0.01] | 0.87 | 0.02 | [0.01;0.03] | 0.001 |
| Log(text network) | -0.01 | [-0.02;0.00] | 0.13 | 0.01 | [-0.00;0.02] | 0.10 |
| Log(Frequency of call interactions) | 0.01 | [-0.02;0.04] | 0.55 | 0.03 | [0.02;0.05] | <0.001 |
| Log(Frequency of text interactions) | 0.00 | [-0.02;0.00] | 0.94 | 0.03 | [0.01;0.05] | 0.01 |
| Log(Call duration) | 0.04 | [0.02;0.06] | 0.00123 | 0.02 | [0.01;0.04] | < 0.001 |
| 95%CI= 95% confidence interval. All estimates adjusted for age, gender and personality | | | | | | |
